# Framingham risk scores for determination the 10-year risk of cardiovascular disease in participants with and without the metabolic syndrome: results of the Fasa Persian cohort study

**DOI:** 10.1186/s12902-024-01621-5

**Published:** 2024-06-24

**Authors:** Azizallah Dehghan, Leila Jahangiry, Rozhan Khezri, Alireza Jafari, Babak Pezeshki, Fatemeh Rezaei, Dagfinn Aune

**Affiliations:** 1 Noncommunicable Diseases Research Center, Fasa University of Medical Sciences, Fasa, Iran; 2 Road Traffic Injury Research Center, Tabriz Health Services Management Research Center, Tabriz University of Medical Sciences, Tabriz, Iran; 3 Health Education and Health Promotion Department, School of Health, Medical Education Research Center, Health Management and Safety Promotion Research Institute, Tabriz University of Medical Sciences, Tabriz, Iran; 4 Student Research Committee, Iran University of Medical Sciences, Tehran, Iran; 5 Department of Health Education and Health Promotion, School of Health, Social Development and Health Promotion Research Center, Gonabad University of Medical Sciences, Gonabad, Iran; 6 Research Center for Social Determinants of Health, Jahrom University of Medical Sciences, Jahrom, Iran; 7 Department of Epidemiology and Biostatistics, School of Public Health, Imperial College London, London, UK; 8 Department of Research, Cancer Registry of Norway, Norwegian Institute of Public Health, Oslo, Norway; 9 Department of Nutrition, Oslo New University College, Oslo, Norway

**Keywords:** Cardiovascular disease, 10-year risk, Framingham risk score, Metabolic syndrome

## Abstract

**Background:**

Metabolic syndrome (MetS) is a cluster of risk factors and the Framingham risk score (FRS) is a useful metric for measuring the 10-year cardiovascular disease (CVD) risk of the population. The present study aimed to determine the 10-year risk of cardiovascular disease using the Framingham risk score in people with and without MetS in a large Iranian cohort study.

**Methods:**

This cross-sectional study was done using the Fasa cohort. Participants aged ≥ 35 years old were recruited to the study from 2015 to 2016. The FRS was calculated using age, sex, current smoking, diabetes, systolic blood pressure (SBP), total cholesterol, and high-density lipoprotein (HDL) cholesterol. MetS was defined as the presence of three or more of the MetS risk factors including triglyceride (TG) level ≥ 150 mg dl^− 1^, HDL level < 40 mg dl^− 1^ in men and < 50 mg dl^− 1^ in women, systolic/diastolic blood pressure ≥ 130/≥85 mmHg or using medicine for hypertension, fasting blood sugar (FBS) level ≥ 100 mg dl^− 1^ or using diabetes medication and abdominal obesity considered as waist circumference (WC) ≥ 88 cm for women and ≥ 102 cm for men. Multiple logistic regressions were applied to estimate the 10- year CVD risk among people with and without MetS.

**Results:**

Of 8949 participants, 1928 people (21.6%) had MetS. The mean age of the participants with and without Mets was 50.4 ± 9.2 years and 46.9 ± 9.1 years respectively. In total 15.3% of participants with MetS and 8.0% of participants without MetS were in the high-risk category of 10-year CVD risk. Among participants with MetS gender, TG, SBP, FBS and in people without MetS gender, TG, SBP, FBS, and HDL showed strong associations with the predicted 10-year CVD risk.

**Conclusion:**

Male sex and increased SBP, TG, and FBS parameters were strongly associated with increased 10-year risk of CVD in people with and without MetS. In people without MetS, reduced HDL-cholestrol was strongly associated with increased 10-year risk of CVD. The recognition of participant’s TG, blood pressure (BP), FBS and planning appropriate lifestyle interventions related to these characteristics is an important step towards prevention of CVD.

## Background

Metabolic syndrome (MetS) is a clustering of metabolic risk factors including elevated fasting blood glucose, abdominal obesity, elevated triglycerides, hypertension, and low high-density lipoprotein cholesterol levels, risk factors that are known to increase the risk of cardiovascular diseases [1]. According to the Centers for Disease Control and Prevention (CDC), the prevalence of MetS in the United States increased by 35% from 1980 to 2012 [2]. The prevalence of MetS among the Iranian population is also increasing. The Tehran Lipid and Glucose Study (TLGS) reported the prevalence of MetS for women at 42% and for men at 24% [3] in an urban population. Another study from Iran on bi-ethnic Turk and Kurd populations aged 30–70 years found that the prevalence of MetS among Turk people was 41.6% and among Kurd People was 33.9% [4]. The high prevalence rate of MetS may be explained by their cultural and lifestyle differences [4].

People with MetS have a higher risk of developing CVDs [5, 6]. The presence of four to five components of the MetS has been associated with a 3.7-fold increase in risk of coronary heart disease (CHD) [7], and has also been associated with increased mortality from CVDs and stroke [8]. The economic burden of these chronic diseases imposes a huge cost on the community, individuals, and health care systems. The cost of CVDs in the United States is estimated at more than $200 billion annually, and is expected to double or triple over the next few decades [9]. CVDs that are currently responsible for 17 million deaths a year are projected to increase by about 30% in the next ten years as well as to increase to 23.6 million by 2030 [10]. CVDs were responsible for 41.3% of all deaths in Iran [11].

Given the high prevalence of CVDs, early detection of people at high risk for CVDs is important. Estimating the 10-year risk of CVD using the FRS is one of the major approaches to predict the risk of CVDs. FRS is the most widely used and validated tool for assessing the 10-year risk of CVDs in people without a history of CVDs. Numerous studies have been performed to determine the 10-year risk of CVD through the FRS [12, 13]. It was shown that the 10-year FRS among people with MetS is higher than people without MetS [13, 14]. In a study on FRS estimation in people with metabolic syndrome, it was shown that 16.3% of people were at moderate risk, and 6.3% were at high risk of developing CVDs over the next 10 years [14]. Since MetS is a cluster of risk factors and FRS as a useful metric for measuring the CVD risk in a population, comparing the estimated 10-year risk of CVDs among persons with and without MetS may be important for risk stratification and could refine strategies for preventing CVDs in the general population. Therefore, the present study aimed to determine the 10-year risk score of Framingham in people with and without MetS in a large sample size.

## Methods

### Study population and design

This cross-sectional study was conducted in southwest Iran and was based on the Fasa cohort study. The study aims to evaluate and monitor risk factors for non-communicable diseases (NCDs). The methodological details of the Fasa cohort have been documented elsewhere [15, 16]. In summary, the Fasa cohort study included 10,138 individuals aged ≥ 35 years old at baseline between 2015 and 2016. Trained research interviewers collected information from the participants using a questionnaire which included information on socio-demographic factors, such as age, gender, education, socio-economic status (SES), lifestyle factors including current smoking status (yes/no), alcohol intake (yes/ no), and medical history including diabetes, hypertension, and CVDs such as CHD, myocardial infarction, and stroke, and clinical measurements (anthropometry, lipids and BP). Height, weight, and BP were measured according to the study protocol. SBP and diastolic blood pressure (DBP) were measured twice for each participant from the right and left arms with appropriate cuffs using a mercury sphygmomanometer after 15 min of rest, and then their average SBP and DBP was recorded in mmHg. A blood sample was drawn after 10–14 h of overnight fasting. Diabetes was defined as fasting blood sugar (≥ 126 mg/dL) or medical records of diabetes. Blood sampling was conducted to measure factors including HDL-cholesterol, LDL-cholesterol, total cholesterol (TC), and triglycerides. All measurements were done according to the PERSIAN cohort guidelines [17]. People who had a history of CVD and stroke were excluded from the study. In total, 8949 persons without CVD or stroke history were included.

### CVD risk calculation

A Framingham risk score calculation was done for the 10-year risk of fatal and non-fatal CVD. The FRS scores were calculated using age, sex, SBP and using the medicine for hypertension, smoking status, diabetes, total cholesterol (mg/dL), and HDL cholesterol (mg/dL) [18].

### Metabolic syndrome

MetS status was determined using the National Cholesterol Education Program (NCEP) Adult Treatment Panel (ATP) III [19]. The presence of three or more of the following risk factors including TG level ≥ 150 mg dl^− 1^, HDL cholesterol level < 40 mg dl^− 1^ in men and < 50 mg dl^− 1^ in women, systolic/diastolic blood pressure ≥ 130/≥85 mmHg or using the medicine for hypertension, FBS level ≥ 100 mg dl^− 1^ or using diabetes medication, and abdominal obesity considered as WC ≥ 88 cm for women and ≥ 102 cm for men, were defined as MetS in the current study.

### Ethical considerations

This study was approved by the Ethics Committee of Jahrom University of Medical Sciences (IR.JUMS.REC.1400.062). All ethical principles including confidentiality and anonymity were considered in information gathering. Informed consent was obtained from all participants.

### Statistical analysis

Categorical variables were reported as percentages and continuous variables were reported as means and standard deviations (SDs). MetS status was first determined in the total population; then FRS was calculated for all individuals with and without MetS. FRSs were classified into three groups: low (< 10%), moderate (10–20%), and high (≥ 20%). Then, FRSs were divided into two groups of < 10% and ≥ 10% risk. In order to determine the associations between 10-year CVD FRS with all variables in participants with MetS, univariate analyses were conducted and crude odds ratios (ORs) were calculated comparing the two groups. Multiple logistic regression analyses were used to estimate adjusted ORs 95% confidence intervals (CIs) for the association between 10-year CVD risk and socio-demographic factors as independent and dependent variables, respectively. The statistical analyses were performed with Statistical Package for Social Science (IBM SPSS Statistics for Windows, Version 23.0. Armonk, NY: IBM Corp). A *p*-value < 0.05 was considered statistically significant.

## Results

Of 8949 participants, 1928 people (21.6%) had MetS. The mean age of participants with MetS was 50.4 ± 9.2 years, and of those without MetS was 46.9 ± 9.1 years. Analyses stratified by sex showed 10.7% of men and 30.9% of women had MetS. The mean of age of individuals with MetS was significantly higher than among those without MetS (Table 1).

**Table 1:** Characteristics of study population

Characteristics	Total *N* = 8944	With MetS *N* = 1928	Without MetS *N* = 7016	*P*-value*
Age (year)	47.7 ± 9.2	50.4 ± 9.2	46.9 ± 9.1	< 0.001
Female gender	4812(53.8)	1485(30.9)	3327(69.1)	< 0.001
SBP (mmHg)	110.5 ± 17.8	120.9 ± 19.5	107.6 ± 16.2	< 0.001
DBP (mmHg)	74.3 ± 11.8	80.3 ± 12.4	72.6 ± 11.0	< 0.001
WC (cm)	92.7 ± 11.7	101.7 ± 9.7	90.3 ± 11.0	< 0.001
BMI (kg/m2)	25.5 ± 4.8	28.9 + 4.4	24.6 ± 4.5	< 0.001
FBS (mg/dl)	91.3 ± 27.5	106.9 ± 44.3	87.1 ± 18.4	< 0.001
TC (mg/dl)	185.6 ± 38.3	197.7 ± 41.2	182.2 ± 36.8	< 0.001
TG (mg/dl)	131.0 ± 82.3	193.7 ± 109.1	113.7 ± 65.2	< 0.001
HDL (mg/dl)	51.0 ± 16.0	45.1 ± 13.1	52.6 ± 16.3	< 0.001
LDL (mg/dl)	108.3 ± 32.2	113.7 ± 35.0	106.8 ± 31.1	< 0.001

*t-test

SBP, systolic blood pressure; DBP, diastolic blood pressure; WC, waist circumference; BMI, body mass index; FBS, fasting blood sugar ; TG, triglyceride; TC, total cholesterol; HDL, high density lipoprotein; LDL, low density lipoprotein

**Fig. 1:**
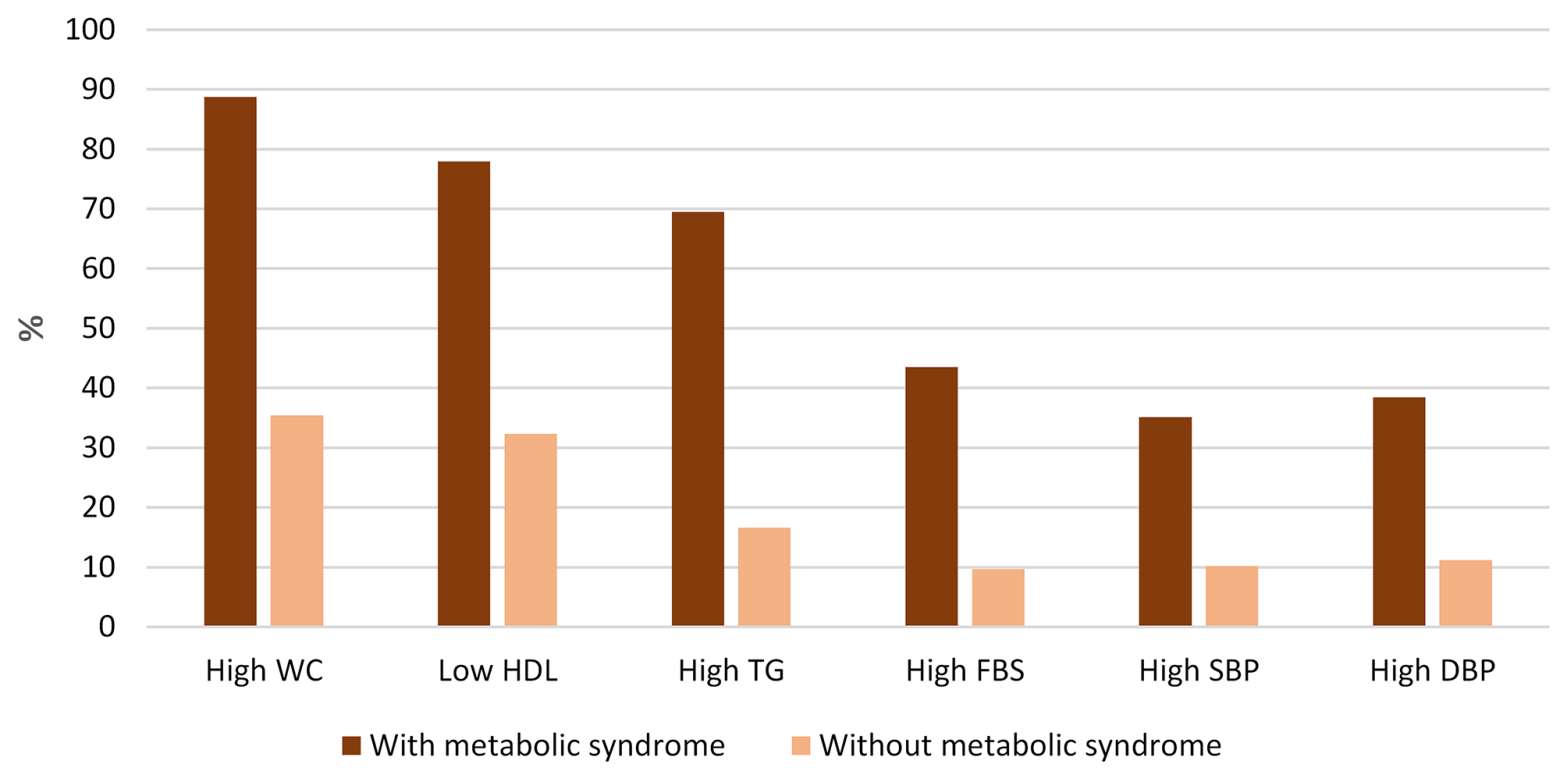
Prevalence of MetS components in the participants with and without metabolic syndrome

The prevalence of individual components of MetS in the participants with and without MetS is shown in Fig. 1. The prevalence of high WC, high TG, high FBS, high DBP, and high SBP was higher in individuals with MetS than among those without MetS, but individuals with MetS had a higher prevalence of low HDL cholesterol.

**Fig. 2:**
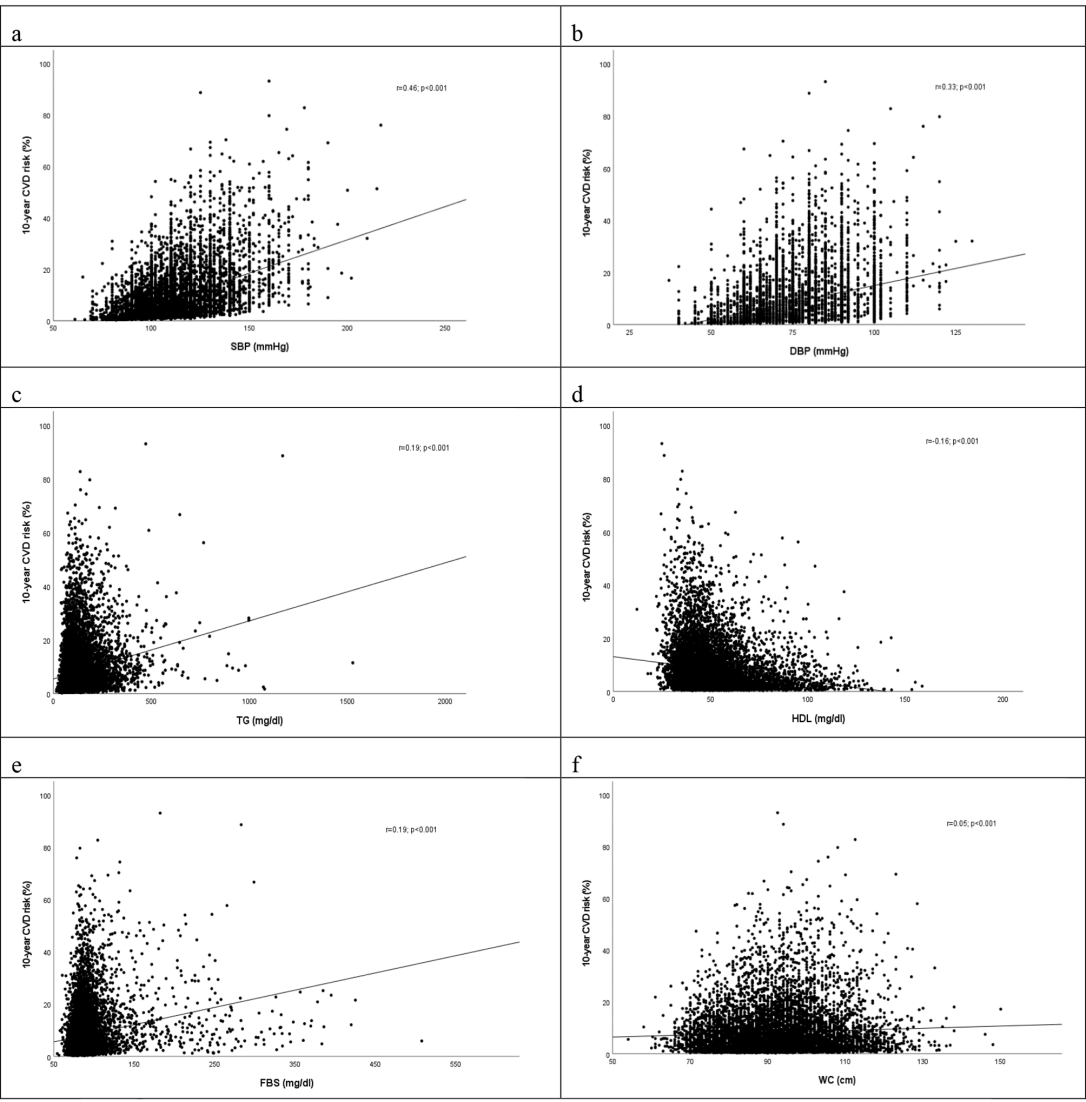
Correlation between 10-year FRS and metabolic syndrome components. **a** SBP, **b** DBP, **c** TG, **d** HDL, **e** FBS, **f** WC

Scatter plots of the correlation between 10-year FRS and MetS components have been shown in Fig. 2. Accordingly, a very weak positive correlation was found amongst 10-year FRS and FBS, TG, and WC. Correlation between 10-year FRS and DBP was positive and weak and between 10-year FRS and SBP was positive and moderate. A very weak negative correlation was found between 10-year FRS and HDL.

The 10-year cardiovascular risk in participants with and without MetS according to their characteristics is showed in Table 2. In total, 63.4% of participants with MetS were in the low-risk category, 21.3% in moderate-risk, and 15.3% in the high-risk category. Also, 75.9% of people without MetS were in the low-risk category, 16.1% in moderate-risk, and 8.0% in the high-risk category. The result showed that 42.7% of men and 7.1% of women with MetS were classified in the high-risk group. The results showed that in participants with MetS there is a significantly difference between 10-year CVD categories by age, sex, SBP, DBP, TG, and FBS (*p* < 0.001). In people without MetS, there is a significantly difference between 10-year CVD categories by age, sex, SBP, DBP, TG, HDL, WC and FBS (*p* < 0.001).

**Table 2:** The 10-year cardiovascular risk in individuals with and without metabolic syndrome according to baseline characteristics

Variables	CVD risk categories							
	With MetS				Without MetS			
	Low risk	Moderate risk	High risk	*P*-value*	Low risk	Moderate risk	High risk	*P*-value*
Age; years (mean ± SD)	46.6 ± 7.8	54.8 ± 7.9	59.5 ± 6.9	< 0.001	44.2 ± 7.6	53.5 ± 7.4	60.2 ± 6.6	< 0.001
Sex, female/male	1097/125	282/129	106/189	< 0.001	3168/2159	139/987	20/543	< 0.001
WC, cm	101.6 ± 9.4	101.2 ± 10.5	102.5 ± 9.9	0.20	90.5 ± 11.3	89.1 ± 10.4	90.6 ± 9.6	0.0003
SBP, mmHg	113.7 ± 15.8	129.4 ± 16.9	138.4 ± 20.9	< 0.001	104.3 ± 13.6	114.0 ± 17.5	126.0 ± 19.9	< 0.001
DBP, mmHg	77.5 ± 11.9	83.7 ± 11.3	87.4 ± 11.7	< 0.001	71.2 ± 10.3	75.3 ± 11.6	80.7 ± 12.1	< 0.001
TC, mg/dl	193.6 ± 38.5	204.3 ± 43.2	205.2 ± 46.6	< 0.001	179.9 ± 36.4	189.2 ± 37.7	190.1 ± 35.2	< 0.001
HDL, mg/dl	44.4 ± 12.5	45.3 ± 12.7	43.5 ± 15.8	0.07	53.8 ± 16.6	49.1 ± 14.7	47.7 ± 13.8	< 0.001
LDL, mg/dl	111.1 ± 33.2	118.0 ± 36.4	118.6 ± 39.2	0.0001	104.2 ± 30.7	114.3 ± 31.8	116.6 ± 29.6	< 0.001
TG, mg/dl	184.7 ± 93.3	204.9 ± 126.9	215.2 ± 135.5	< 0.001	109.1 ± 57.0	128.2 ± 75.7	128.5 ± 81.8	< 0.001
FBS, mg/dl	100.4 ± 37.1	115.0 ± 50.8	122.4 ± 54.9	< 0.001	85.8 ± 15.4	89.7 ± 25.1	92.6 ± 25.6	< 0.001

*One way ANOVA

When categorizing 10-year CVD risk into two groups (< 10%, and ≥ 10%), predictors of CVD risk were assessed by multiple analyses for individuals with MetS in Table 3 and for individuals without MetS in Table 4. Among participants with MetS, men [OR 11.8, 95% CI: 8.4–16.4; *p* < 0.001], high SBP [OR 11.2, 95% CI: 8.2–15.3; *p* < 0.001], high TG [OR 1.7, 95% CI: 1.3–2.3; *p* < 0.001], and high FBS [OR 6.1, 95% CI: 4.6–8.0; *p* < 0.001] showed strong and significant associations with higher 10-year CVD risk. Among individuals without MetS, men [OR 29.3, 95% CI: 22.5–38.0; *p* < 0.001], high SBP [OR 16.8, 95% CI: 12.5–22.5; *p* < 0.001], low HDL [OR 1.4, 95% CI:1.2–1.6; *p* < 0.001], high TG [OR 1.3, 95% CI:1.1–1.5; *p* = 0.001], and high FBS [OR 4.8, 95% CI: 3.9–6.0; *p* < 0.001] showed strong and significant associations with higher 10-year CVD risk.

**Table 3:** Univariable and multiple logistic regression results with potential determinants of elevated CVD risk in individuals with metabolic syndrome

Variables	CVD risk < 10% *N* = 1222	CVD risk ≥ 10% *N* = 706	Univariable Logistic Regression		Multiple Logistic Regression		
	*N* (%)	*N* (%)	COR (95% CI)	*P*-value	AOR (95% CI)	*P*-value	
**Sex**							
Female	1097 (73.9)	388 (26.1)	Ref.	Ref.	Ref.	Ref.	
Male	125 (28.2)	318(71.8)	7.2 (5.7–9.1)	< 0.001	11.8 (8.4–16.4)	< 0.001	
**WC, cm**							
Normal	63 (29.0)	154 (80.0)	Ref.	Ref.	Ref.	Ref.	
High	1159 (67.7)	552 (32.3)	0.19 (0.14–0.26)	< 0.001	0.79 (0.52–1.21)	0.28	
**SBP, mmHg**							
Normal	971 (77.6)	281 (22.4)	Ref.	Ref.	Ref.	Ref.	
High	251 (37.1)	425 (62.9)	5.8 (4.7–7.2)	< 0.001	11.2 (8.2–15.3)	< 0.001	
**DBP, mmHg**							
Normal	853 (72.2)	328 (27.8)	Ref.	Ref.	Ref.	Ref.	
High	369 (49.4)	378 (50.6)	2.7 (2.2–3.2)	< 0.001	1.1 (0.84–1.5)	0.45	
**HDL, mg/dl**							
Normal	213 (50.0)	213 (50.0)	Ref.	Ref.	Ref.	Ref.	
Low	1009 (67.2)	493 (32.8)	0.48 (0.39–0.61)	< 0.001	1.1 (0.85–1.5)	0.36	
**TG, mg/dl**							
Normal	378 (64.4)	209 (35.6)	Ref.	Ref.	Ref.	Ref.	
High	844 (62.9)	497 (37.1)	1.1 (0.87–1.3)	0.54	1.7 (1.3–2.3)	< 0.001	
**FBS, mg/dl**							
Normal	802 (73.6)	287 (26.4)	Ref.	Ref.	Ref.	Ref.	
High	420 (50.1)	419 (49.9)	2.8 (2.3–3.4)	< 0.001	6.1 (4.6-8.0)	< 0.001	

**Table 4:** Univariable and multiple logistic regression results with potential determinants of elevated CVD risk in individuals without metabolic syndrome

Variables	CVD risk < 10% *N* = 5327	CVD risk ≥ 10% *N* = 1689	Univariable Logistic Regression		Multiple Logistic Regression		
	*N* (%)	*N* (%)	OR (95% CI)	*P*-value	OR (95% CI)	*P*-value	
**Sex**							
Female	3168 (95.2)	159 (4.8)	Ref.	Ref.	Ref.	Ref.	
Male	2159 (58.5)	1530 (41.5)	14.1 (11.9–16.8)	< 0.001	29.3 (22.5–38.0)	< 0.001	
**WC, cm**							
Normal	3065 (67.6)	1466 (32.4)	Ref.	Ref.	Ref.	Ref.	
High	2262 (91.0)	223 (9.0)	0.21 (0.17–0.24)	< 0.001	1.2 (0.97–1.52)	0.088	
**SBP, mmHg**							
Normal	5055 (80.3)	1242 (19.7)	Ref.	Ref.	Ref.	Ref.	
High	272 (37.8)	447 (62.2)	6.7 (5.7–7.9)	< 0.001	16.8 (12.5–22.5)	< 0.001	
**DBP, mmHg**							
Normal	4915 (78.9)	1313 (21.1)	Ref.	Ref.	Ref.	Ref.	
High	412 (52.3)	376 (47.7)	3.4 (2.9-4.0)	< 0.001	1.2 (0.92–1.5)	0.19	
**HDL, mg/dl**							
Normal	3560 (74.9)	1192 (25.1)	Ref.	Ref.	Ref.	Ref.	
Low	1767 (78.0)	497 (22.0)	0.84 (0.74–0.94)	0.004	1.4 (1.2–1.6)	< 0.001	
**TG, mg/dl**							
Normal	4554 (77.9)	1295 (22.1)	Ref.	Ref.	Ref.	Ref.	
High	773 (66.2)	394 (33.8)	1.8 (1.6-2.0)	< 0.001	1.3 (1.1–1.5)	0.001	
**FBS, mg/dl**							
Normal	4934 (77.9)	1398 (22.1)	Ref.	Ref.	Ref.	Ref.	
High	393 (57.5)	291 (42.5)	2.6 (2.2-3.0)	< 0.001	4.8 (3.9-6.0)	< 0.001	

## Discussion

The present cross-sectional study was conducted in a large Iranian population to determine 10-year Framingham risk scores in people with and without MetS. The results showed that 36.6% of participants with MetS and 24.1% people without MetS were in moderate- and high-risk CVDs over the next 10 years. In this study, the prevalence of MetS in the total population was 21.6%. The findings of our study were consistent with other studies conducted in Iran so the prevalence of MetS in other studies has been reported from 10 to 47% [13, 20–22]. The prevalence of MetS in neighboring countries has been reported between 2.2% and 63% [23]. The wide variety in the prevalence of MetS in different parts of the world may be explained by differences in geographical, ethnic, racial, and lifestyle factors.

In this study, the prevalence of MetS was higher in women than men (30.9% vs. 10.7%). The prevalence of Mets increases with age [24]. Due to women after menopause and to a rapid impairment in endothelial function have a dramatic increase in BP [25]. There is sharper age-related increase in MetS prevalence in women than men [26].

The results showed that 15.3% of participants with MetS and 8.0% of people without MetS were at a high risk of 10- years of FRS. Other studies have shown that people with MetS have a higher risk of cardiovascular disease [5, 6]. Therefore, appropriate and opportune interventions [21, 27] and strategies are needed to prevent CVD in people with Mets.

To determine the risk factors of 10-year CVD in people with and without MetS, we divided the scores into < 10% and ≥ 10%. In people with MetS, age was associated with an increased 10-year risk of CVD. Age was a risk factor for the 10-years risk of FRS. Yazdanyar et al. showed that higher age was associated with increased prevalence of CVDs including atherosclerosis, stroke, and myocardial infarction in males and females [28]. Age has a critical role in impaired cardiovascular function, which increases the risk of CVD in older people [29, 30].

The results showed that both men with and without MetS were at strongly increased 10-year CVD risk. In a study by Farhangi et al., men had a higher risk of CVD when compared with women [31]. Yousefzadeh et al. showed that for 10-year FRS CVD risk was higher in men than in women [13] and similar results were reported by Deepali et al. [32]. Therefore, gender differences in awareness is important for preventing and managing CVD [33].

The results showed that in people with and without Mets, the 10-year risk of CVD was higher in people with high SBP. In a prospective study by Ma et al. a strong positive association was reported between mean SBP and CVD risk [34]. Duangjai et al., showed that higher SBP and DBP was associated with a strong positive association with CVD risk prediction score [35]. The study by Liszka et al. also found a positive association between BP and risk of CVDs [36], including arteriosclerotic disease, congestive heart failure and cerebrovascular disease. However, treatment of high BP is very important. But still, there is inadequate control and treatment for many people with high BP. Lack of patient knowledge, cost and barriers of treatment, and the asymptomatic nature of hypertension may be related to inadequate control of hypertension [37].

In this study, high TG was associated with increased 10-year risk of CVD. Gu et al. reported that elevated levels of serum TG were positively associated with risk of ischemic stroke [38]. Brunner et al. showed that TG has been recognized as a risk factor for CVD [39]. So, in people with hypertriglyceridaemia, lifestyle changes and drug therapy are important factors to consider to prevent CVD.

According to the results, individuals with high FBS compared with those who had normal FBS had a higher 10-year risk of CVD. A study by Wu et al. found that significant association between high FBS with all-cause, CVD, and expanded CVD mortality [40]. He et al., showed that diabetes is one of the most important risk factors for CVD [41]. So, the treatment of patients with diabetes is essential for the prevention of CVD death and disability.

The results showed that only in people without MetS, low HDL-cholesterol was associated with 10-year risk of CVD. Cooney et al., showed that there was a strong and inverse association between HDL- cholesterol and CVD and CHD mortality [42]. The study by Santos-Gallego et al. also found an inverse association between HDL-cholesterol and CVD risk [43]. Barter et al. shown that there is an inverse association between serum HDL-cholesterol levels and the risk of CHD [44].

The results showed that in people with MetS, measures of central obesity showed no association with 10-year risk of CVD, however, it is likely that the impact of central obesity on CVD risk at least partly is mediated through lipids, elevated BP, and blood glucose, and the current study may have over-adjusted by including intermediate factors in the multiple model. Duangjai et al., showed that there was a strong positive association between WC and CVD risk prediction score [35]. Also, body mass index (BMI) has a strong association with higher prevalence of CVD independent of MetS [41]. Other studies have shown the combination of abnormal BMI and WC is more likely to predict the risk of CVD [45, 46]. However, in this population-based study there was conflicting evidence on the association of high WC with CVD risk.

This study is the first study in a large Iranian population to compare the 10-year risk of CVD in people with and without MetS. The current results would be generalizable to a similar population. One of the limitations of the study is its cross-sectional design, which cannot infer causality, and further prospective studies are needed in this population. In addition, although the Framingham risk score is a useful tool for determining the 10-year risk of CVD, it cannot be considered as a completely accurate tool at the individual level, and medical and clinical examinations must be performed to confirm it.

## Conclusion

The results showed that in people with and without MetS, male sex and increased SBP, TG, and FBS were risk factors strongly associated with increased 10-year predicted risk of CVD. In people without MetS, decreased HDL-cholestrol was strongly associated with increased 10-year predicted risk of CVD. The recognition of these risk factors in primary care centers and planning appropriate lifestyle interventions is of major importance for CVD prevention. Further longitudinal studies are needed on the association between MetS components and the 10-years risk of CVD in this population.

## Data Availability

All data generated or analysed during this study are included in this published article.

## Abbreviations

MetS: Metabolic syndrome
FRS: Framingham risk score
CVD: Cardiovascular disease
SBP: Systolic blood pressure
DBP: Diastolic blood pressure
BP: Blood pressure
HDL: High-density lipoprotein
LDL: Low-density lipoprotein
TG: Triglyceride
TC: Total cholesterol
FBS: Fasting blood sugar
WC: Waist circumference
BMI: Body mass index
NCDs: Non-communicable diseases
SES: Socio-economic status
CHD: Coronary heart disease
SD: Standard deviation
OR: Odds ratio
CI: Confidence interval
